# Stability and reliability of perovskite photovoltaics: Are we there yet?

**DOI:** 10.1557/s43577-025-00863-5

**Published:** 2025-03-18

**Authors:** Kenedy Tabah Tanko, Zhenchuan Tian, Sonia Raga, Haibing Xie, Eugene A. Katz, Monica Lira-Cantu

**Affiliations:** 1 Catalan Institute of Nanoscience and Nanotechnology (ICN2), CSIC and Barcelona Institute of Science and Technology, Bellaterra, Barcelona, Spain; 2 Institute for Advanced Study, Shenzhen University, Shenzhen, China; 3 Department of Solar Energy and Environmental Physics, Swiss Institute for Dryland Environmental and Energy Research, J. Blaustein Institutes for Desert Research, Ben-Gurion University of the Negev, Midreshet Ben-Gurion, Israel

**Keywords:** Stability of perovskite solar cells, ISOS protocols, Commercialization, Industrial modules, Accelerated tests

## Abstract

**Abstract:**

The power-conversion efficiency (PCE) of perovskite solar cells (PSCs) has exceeded in 2024 the theoretical single-junction Shockley–Queisser limit of 33.7% with the perovskite/silicon tandem version. The commercialization of the technology is now a reality with the PV industry demonstrating its first commercial products. Many companies have shown excellent module reliability with most of them passing the IEC standardization (required for commercial silicon solar cells). In this article, we want to bring some light on the most intriguing question regarding the stability and reliability of PSC technology: Are we there yet? Issues on stability are still under strong investigation and research on the topic has increased exponentially in the last 10 years. Since some companies have already promised excellent reliability of their modules, with 80% retention of the initial PCE after 25 years, the following  two or three years will be crucial to demonstrate these pledges. In this work, we present an outline of the most stable PSC devices reported to date and discuss the most important strategies leading to highly stable devices.

**Graphical abstract:**

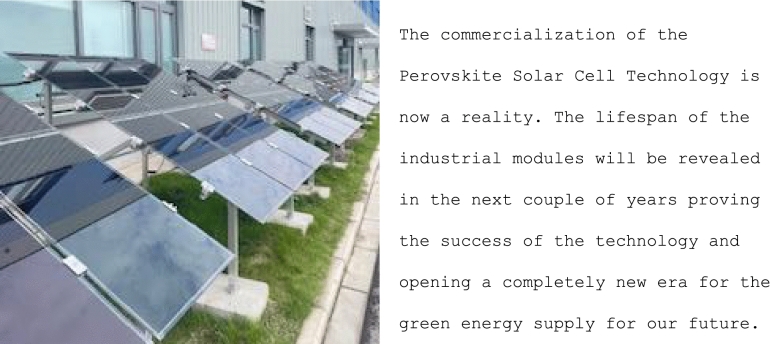

**Supplementary information:**

The online version contains supplementary material available at 10.1557/s43577-025-00863-5.

## Introduction

Perovskite solar cells (PSCs) and modules (PSMs) exhibit meteoritic growth in their power-conversion efficiency (PCE) reaching 26.7% and 34.7% for single-junction and tandem (with Si) configurations, respectively. PSC technology has, in 2024 and for the first time, surpassed the single-junction Shockley–Queisser limit of 33.7% PCE.^1^ Investments in the technology have reached billions of Euros^2^ and the commercialization of the technology is now a reality. Companies such as RenShine Solar, GCL-Si, LONGi, or Oxford PV, among many others, have announced the commercialization of their PSM. Oxford PV has informed the commercialization of their tandem perovskite-silicon PSM with PCE of 24 percent.^3^ The world’s first commercial gigawatt-scale manufacturing of PSCs (1.2 m × 2.4 m module size) was announced in December 2023 by GCL Solar Energy for their single-junction PSCs.^4^ RenShine Solar announced in 2024, their 1.2 megawatt-scale perovskite distributed power station connected to the grid.^5^ PV industries are also promising very competitive module reliability with most passing the International Electrotechnical Commission (IEC) standardization (compulsory for commercial silicon solar cells).

In this article, we want to bring some light on the most intriguing question about the stability of PSC technology, its laboratory-scale stability and commercial-module reliability: Are we there yet? Although efficiency values have reached astonishing values, issues on stability are still under investigation and research on the topic has increased exponentially in the last 10 years. Some companies are already promising excellent reliability of their PSM retaining 80% of the initial PCE after 25 years.^6^ Confirming these promises will be crucial in the next two or three years for the success of this PV technology. In this work, we present an outline of the most stable PSC devices reported to date, Pb-free PSC and commercial PSCs in both single-junction and tandem configurations. We will discuss the most important strategies leading to highly stable devices. We will focus our attention on the ISOS protocols for stability testing, especially International Summit on Organic and Hybrid Photovoltaics Stability (ISOS) protocols under light irradiation conditions (ISOS-L) and outdoor stability assessment (ISOS-O).^7^

**Pb-containing perovskites.** The presence of 6*s*2 (5*s*2) lone-pair electrons on the *B*-site Pb (also Sn) in hybrid halide *ABX*3 perovskites is fundamental to many of their appealing and special optoelectronic properties. This lone pair leads to mobile holes, favorable bandwidths and band alignments, large ionic dielectric response, and large positive thermal expansion. This lone-pair electron configuration gives halide perovskite its defect tolerance nature and affects lattice dynamics and large ionic dielectric response, among many other properties.^9^ The BX_6_ octahedron framework that constitutes the metal halide perovskite crystal structure ABX_3_ is believed to be mainly responsible for the structural instability of halide perovskite materials. Ion migration along the PbI_6_ octahedron rules this stability, because the iodide ion has the lowest activation energy for ion migration, followed by the MA^+^ cation.^10–12^ These mobile iodide ions are also known for their easy oxidation and can decompose into bimolecular iodine by photogenerated holes. As a result, Pb defects are formed with time, leading to irreversible phase separation and halide perovskite degradation.^13–15^

**Figure 1:**
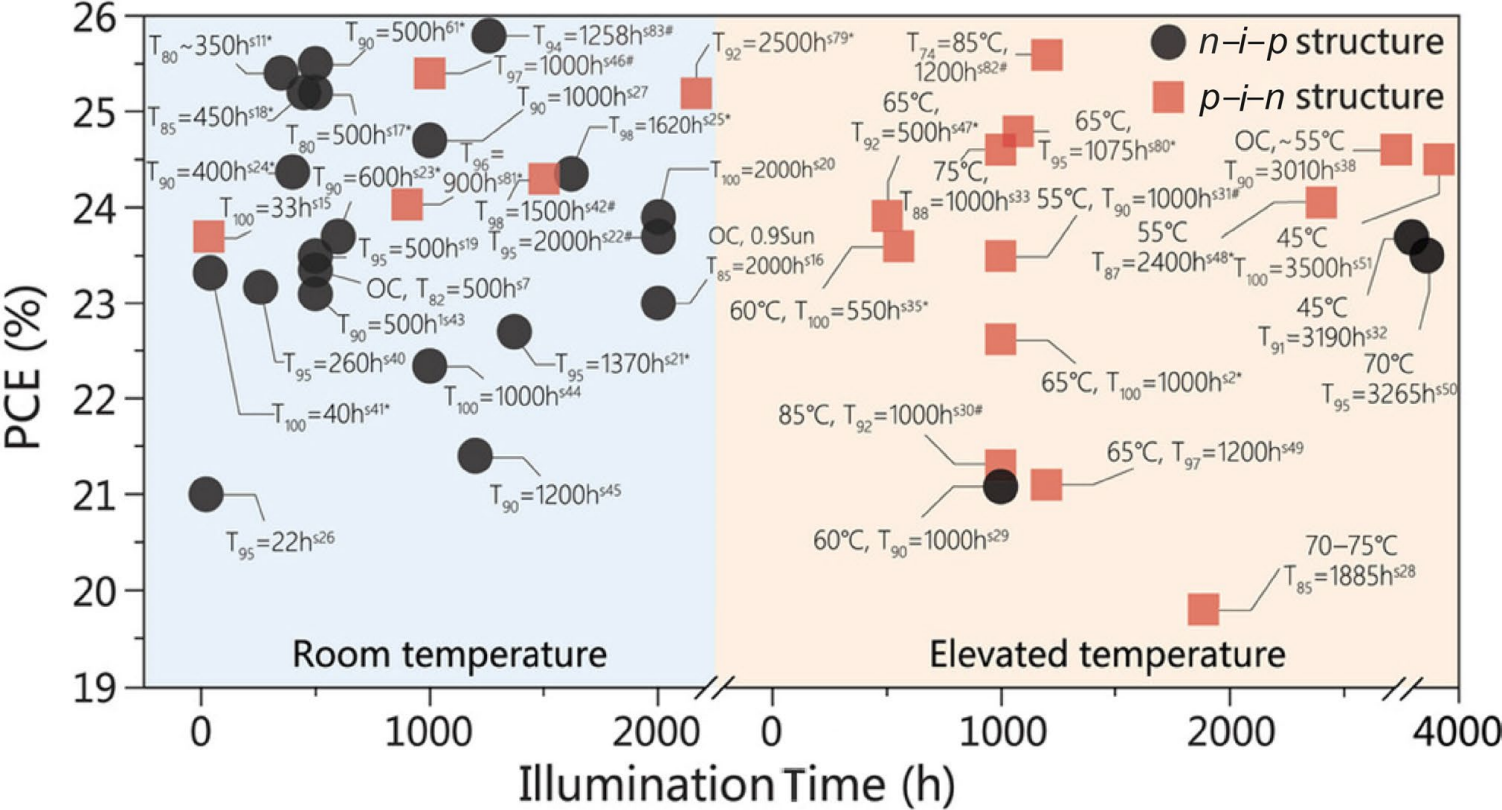
Evolution of efficiency and stability of perovskite solar cells. Typical stability of perovskite solar cells under continuous illumination. Txx = Y h means that the power-conversion efficiencies (PCEs) drop to xx% of the initial value after Y-hour aging. Reprinted with permission from Reference 8. © 2024 Wiley.^8^

**Indoor stability. ISOS-L. Figure** **1** summarizes the recently published data for PSCs, in inverted and normal configurations.^8^ The data correspond to the stability under continuous illumination, which is one of the most important PSC stability tests. To complement this graph,** Table** **I** displays the most recent stability reports for PSCs for ISOS-L protocol, published only in 2024.

**Table I:** Indoor stability analysis (for published PSCs in 2024 only).

Structure	PCE (%)	Light Intensity	Temp (°C)	Tracking Method	Test Time (h)	PR (%)	Reference
FTO/SnO_2_/(FAPbI_3_)_0.95_(MAPbBr3)_0.05_/Polyspiro/Spiro-OMeTAD/Au	24.54	1 sun	23	MPP	1250	95	22
FTO/c-TiO_2_/m-TiO_2_/Perovskite/Spiro-OMeTAD/Au	23	1 sun	r.t	MPP	4500	99	23
ITO/SnO_2_/FAPbI_3_/HTL (SBF-FC)/Au	25.3	1 sun	65	MPP	500	92	24
ITO/P3CT-N/CsPbI_2_Br/PCBM/C_60_/2,9-dimethyl-4,7-diphenyl-1,10-phenanthroline (BCP)/Ag	15.92	1 sun	45	MPP	500	81.8	25
Glass/ITO/SnO_2_/Perovskite/C_60_/BCP/Ag	25	1 sun	40	MPP	1000	90	21
ITO/NiOx/2PACz/Perovskite/PCBM/BCP/Cr/Au	24.6	1 sun	45	MPP	1000	82	26
Glass/FTO/SnO_2_/Perov/Spiro-OMeTAD/Ag	24.6	1 sun	65	MPP	1920	88	27
FTO glass/SnO_2_/Perov/Spiro-OMeTAD/Ag	24.57	1 sun	rt	MPP	1000	92	28
Glass/Gold/SnO_x_/Perov-Perov/NiO_x_/FTO/glass	21	1 sun	40	*V*_oc_	1000	75	29
FTO/NiO_x_/Me–4PACz/Perovskite/C60/ALD–interlayer/Cu	24.6	1 sun	65	MPP	1350	88	30
FTO/SnO_2_/Perovskite/B(CE)ACl or CEACl/Spiro-OMeTAD/Au	25.6	1sun	55	MPP	500	91	31
ITO/MeO-2PACz/Perovskite/Phenethylammonium iodide (PEAI)/C_60_/ZnO/Au	23.2		85	MPP	1200	98.2	19
ITO/MeO-2PACz/Cs_0.05_MA_0.10_FA_0.85_PbI_3_/PI/C_60_/bathocuproine/Ag	25.07	1 sun	rt	MPP	1000	95	32
FTO/TiO_2_/DDE/MAPbI_3_/Spiro-OMeTAD/Ag	22.1	1 sun	rt	OC	1000	90	33
FTO/SnO_2_/Perovskite/Spiro-OMeTAD/MoO_3_/ITO	223	1 sun	rt	MPP	2000	90	34
FTO/c-TiO_2_/m-TiO_2_/Perovskite/Spiro-OMeTAD/Au	22.3	1 sun	rt	OC	720	82	35
ITO/NiO_x_/Me-4PACz/Perovskite/C_60_/Bathocuproine (BCP)/Cu	22.1	1 sun	rt	MPP	500	100	36
ITO/SnO_2_/PCBM/Cs_0.05_FA_0.85_MA_0.1_Pb(I_0.95_Br_0.05_)_3_/PTAA/BCF/Au	21.3	0.84 sun	85	MPP	937	75	20
Glass/ITO/NiO_x_/Me- 4PACz/WBG perov./C_60_/BCP/Ag	22.2	1 sun	rt	MPP	985	90	37
FTO/SnO_2_/Perovskite (FAPbI_3_)_0.95_(MAPbBr_3_)_0.05_/Poly-v-Spiro/Spiro-OMeTAD/Au	24.5	1 sun	rt	MPP	1250	95	38
ITO/SnO_2_/Perov. (PTAFAPbI_x_Cl_4-x_)/Spiro-OMeTAD/Au	24.5	1 sun	rt	MPP	1000	90	39
ITO/SnO_2_/Perov. (PTAFAPbI_x_Cl_4-x_)/Spiro-OMeTAD/Au	24.5	1 sun	85	MPP	200	97	39
ITO/2PACz/CsFAPbI_3_/C_60_/SnO_2_/IZO/Cu	25.6	1 sun	85		1000	95	21
ITO/NiOx/2PACz/Perovskite without or with 1,3‐PDI or P(1,3‐PDI) additive/PCBM + C_60_/BCP/Cr/Au	24.7	1 sun	45	MPP	1000	82	26
FTO/AI-HAc-SnO_2_ (ETL)/PVK/PCBM/Ag	22.2	1 sun		MPP	1000 h	90	40
Glass/FTO/SnO_2_/Perovskite/Spiro-OMeTAD/Ag	24.6	1 sun	65	MPP	1920	88	27
Glass/FTO/SnO_2_/Perovskite/Spiro-OMeTAD/Ag	24.6	1 sun	65	LC 12 h on/off	10,000	98	27
Glass/ITO/SnO_2_/Perovskite/PC61BM/Au	25.3	1 sun	rt	MPP	1000	85	41
Glass/FTO/SnO_2_/Perovskite/Polymer interlayer/Spiro-OMeTAD/Au	17.8	0.5 sun	65	OC	1440	94	42

PR, performance retention; MPP, max power point tracking; OC, open-circuit voltage.

Another important stability test under constant illumination encompasses high temperatures. Real outdoor conditions of PSCs can reach up to 85°C in some locations during summer at 1 sun (noon). 85°C is also the temperature mark for the standardization of commercial PV devices as stated in the IEC 61215 standard. For PSCs, we have identified no more than eight published works (See Table I and Figure 1) where the stability of PSCs is reported under these extreme conditions of continuous light irradiation of 1 sun and 85°C (ISOS-L2).^16–21^ Among them, the most recent and most robust devices include inverted PSCs and the use of self-assembly monolayers (SAMs) containing carbazole units and methoxysilane or phosphonate anchoring groups. For example, Li and Zhu et al. report the fabrication of inverted PSCs with 25.6% efficiency and 1.53 *V*_oc_, able to maintain 90% of the initial efficiency after 1200 h of continuous light irradiation of 1 sun at 65°C. The authors employ the SAM molecule (4-(3,11-dimethoxy-7H-dibenzo[c,g]carbazol-7-yl)butyl)phosphonic acid (MeO-4PADBC), which is located at the NiO_x_/perovskite interface creating a strong bond of the MeO-4PADBC with the oxide. The authors report that the favorable contact and energetic alignment of the NiO/MeO-4PADBC/perovskite reduces the voltage deficit leading to strong interface toughening effects under thermal stress.^18^ Tang et al. report an inverted PSC with 23.2% efficiency able to retain 98.2% of the initial PCE after 1200 h under continuous light irradiation and 85°C (ISOS-L2). This impressive result has also been attributed to the use of self-assembly monolayers (SAMs) employed as hole-transport layer (HTL). The authors observed that strong polar solvents in the perovskite precursor desorb the SAM if it is anchored on substrates by hydrogen-bonded, rather than covalently bonded, hydroxyl groups. Thus, they created a strong bond between the SAM molecule and a thin layer of indium tin oxide (ITO) deposited by atomic-layer deposition (ALD). The SAM molecule anchors covalently with the oxide substrate via a trimethoxysilane group that exhibited strong tridentate anchoring to the substrate.^19^

J. Luo et al. reported a normal configuration PSC of the type ITO/SnO_2_/PCBM/Cs_0.05_FA_0.85_MA_0.1_Pb(I_0.95_Br_0.05_)_3_/PTAA/BCF/Au with a 21.3% efficiency analyzed under ISOS-L2 protocol and able to retain 75% of the initial efficiency after 937 h of testing under continuous light irradiation at 85°C.^20^ The key of this stable PSC resides in the use of the poly[2,2″″-bis [[(2-butyloctyl)oxy]carbonyl] [2,2′:5′,2″:5″,2″′-quaterthiophene]-5,5″′-diyl] (PDCBT) and tris(pentafluorophenyl)borane (BCF), in a PDCBT/BCF/Au structure, which permits the formation of a robust quasi-ohmic contact boosting the hole injection process from the polymer into metal by two orders of magnitude.^20^ DeWolf et al. report the use of SAM molecules of the type ((2-(9H-carbazol-9-yl)ethyl)phosphonic acid) (2PACz) in an inverted PSC of the type ITO/2PACz/CsFAPbI_3_/C_60_/SnO_2_/IZO/Cu with a PCE of 25.6% (certified 25.0%), which stands 1000 h under continuous light irradiation and 85°C (ISOS-L2) retaining 95% of the initial efficiency after analysis. The key of this PSC resides in the use of double-sided 2D/3D perovskite heterojunctions and the SAM molecules. Long alkyl amine ligands can generate near-phase-pure 2D perovskites at the top and bottom of 3D perovskite interfaces. At the rear-contact side the alkyl amine ligand strengthens the interactions with the substrate through acid–base reactions with the phosphonic acid group from the organic hole-transporting self-assembled monolayer molecule, thus regulating the 2D perovskite formation.^21^

Most of these stable PSCs contain methoxysilane or phosphonate anchoring groups as SAM molecules working as HTL. These functional groups bond strongly with the oxide of the transport layer of the PSC. However, the use of molecules as additives or SAM molecules with strong anchoring groups such as phosphonates have also been reported to be of high importance for the effective immobilization of the halide ions of the perovskite absorber (in the bulk), improving device stability.^43–45^ Several groups, including our group, reported the strong interaction of phosphonate groups with the halide perovskite though hydrogen bonds and the effective passivation of shallow defects such as iodine.^18^ In our case, the phosphonate-based additive employed is a simple molecule added to the bulk of the halide perovskite as an additive with excellent stability at 25°C. Despite these favorable results, our PSCs didn’t pass the stability analysis at high temperatures. On the contrary, SAM molecules with phosphonate anchoring groups and a carbazole core, seem to be the key factor driving the high temperature stability of PSCs samples previously described. This SAM structure is effective when the SAM molecule bonds strongly to an oxide such as the NiO HTL. The latter was observed for PSCs in both single-junction^46,47^ and perovskite-based tandem solar cell configurations.^18,48–52^ In addition, the variation of temperature during the ISOS-L tests are a useful tool employed for the prediction of the PSC lifetime under outdoor or standard test conditions (STC, 1 sun, 25°C) (see the section “Accelerated aging”).

**Outdoor stability. ISOS-O.** A major challenge for PSCs is improving their operational stability under real outdoor conditions that should be monitored under ISOS-O protocol. Besides the practical complication of device encapsulation for outdoor analysis,^53^ the PSC devices have to sustain multiple stressors with great variability and doses: day/night cycling, different “years” seasons (weather, sun irradiance, and temperature), detrimental reverse bias voltage under some cloudy or shadow conditions, among others.^54^ The reports of outdoor stability analyses are very scarce in the literature but highly important for the success of these new commercial PSCs.** Table** **II** encompasses the most important (with respect to testing time) outdoor stability analysis found in the literature to date.

**Table II:** Outdoor stability of PSCs (ISOS-O protocol).

Year	Structure	Initial PCE (%)	Test Mode	Test Time (h)	PR (%)	Reference
*p-i-n*						
2024	ITO/2PACz (SAM)/Cs_0.15_FA_0.85_PbI_2.55_Br_0.45_/C_60_/SnO_2_/Cu	16	MPP	21,900	100	55
2023	Glass/ITO/MeO-2PACz/Rb_0.05_Cs_0.05_MA_0.05_FA_0.85_Pb(I_0.95_Br_0.05_)_3_/C_60_/SnO_2_/Ag	25.5	MPP	4368	65	56
2023	Glass/ITO/MeO-2PACz/FAPbI_3_/LiF (1 nm)/C_60_/BCP/Cu	22.35	MPP	8760	74	57
2021	ITO/HTL/3D/2D PVK/C_l6_SubPc/C_60_/BCP/Ag	19.1	OC	1728	87	58
2020	Glass/ITO/MeO-2PACz/Perovskite/C_60_/SnO_2_/Cu	18.5	MPP	140	100	59
2022	ITO/2PACz (SAM)/Cs_0.15_FA_0.85_PbI_2.55_Br_0.45_/C_60_/SnO_2_/Cu	16	MPP	2880	20	60
2020	FTO/NiOx/Perovskite/PCBM/ITO	12.5	OC	2100	100	61
2019	ITO/NiOx/m-Al_2_O_3_/MAPbI: CH_3_NH_3_PbI_3_/PCBM/Rhodamine/Ag	9.6		2000	70	62
*n-i-p*						
2016	FTO/c-TiO_2_/m-TiO_2_/Perovskite/Spiro-OMeTAD/Au	19	OC	2160	95	63
2021	FTO/c-TiO_2_/m-TiO_2_/Cs_0.5_(MA_0.17_FA_0.83_)_0.95_Pb-(I_0.83_Br_0.17_)_3_/Spiro-OMeTAD/Au	18.5	MPP	500	90	64
2018	Glass/ITO/TiO_2_/MAPbI_3_/Spiro-OMeTAD/Au	15	OC	336	60	65
2020	FTO/c-TiO_2_/m-TiO_2_/ZrO_2_/Perovskite/Carbon	15.6	OC	3600	100	66
2019	Plastic/ITO/SnO_2_/CH_3_NH_3_PbI_3 -x_Cl_x_/Spiro-OMeTAD/Ag	12.7	OC	10	0	67
2015	FTO/c-TiO_2_/m-TiO_2_/ZrO_2_/MAPbI_3_/Carbon	8.2	OC	168	100	68
2016	FTO/c-TiO_2_/m-TiO_2_/FAPbI_3_(0.85)MAPbBr_3_(0.15)/Spiro-MeOTAD/Au	11	OC	1080	60	69
2020	FTO/TiO_2_/FA_0.55_MA_0.25_Cs_0.2_PbI_3_/P3HT/Carbon	18.3	OC	504	37	70
2020	FTO/c-TiO_2_/m-TiO_2_/ZrO_2_/Perovskite/Carbon	6	OC	720	100	71
2022	FTO/c-TiO_2_/mp-TiO_2_/CH_3_NH_3_PbI_3-x_Cl_x_/GC@CH_3_NH_3_PbI_3 -x_Cl_x_/C	5.88	OC	81	81	72
2023	FTO/c-TiO_2_/m-TiO_2_/HP/MXene:H3pp/Spiro-OMeTAD/Au	19.85	MPP	350	60	73
2016	FTO/c-TiO_2_/HP/Perovskite/Spiro-OMeTAD/MoO_3_/Al	15.3	OC	432	72	74

PR, performance retention.

**Figure 2:**
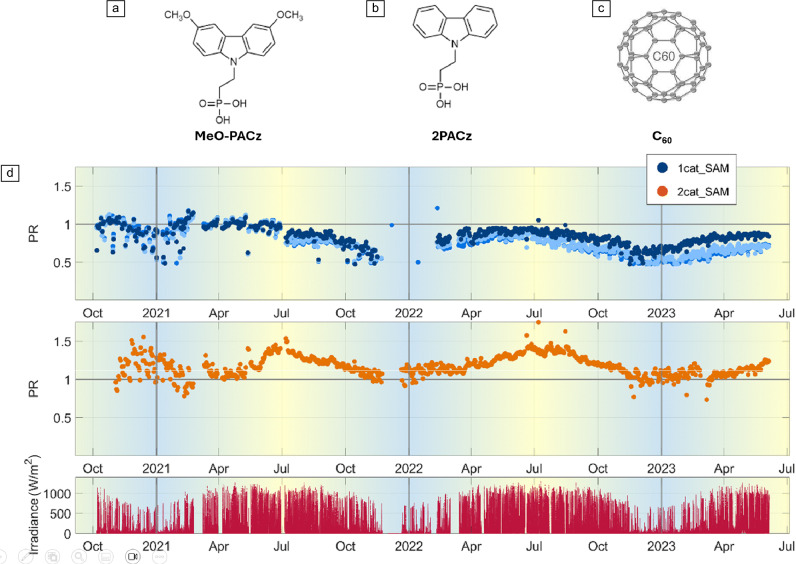
One of the longest outdoor stability assessments reported to date for perovskite solar cells (PSCs). (a–c) The molecules applied as self-assembly monolayers (SAMs) and the electron-transport material employed in the device. (d) The long-term outdoor data for 2.5 years of continuous operation of laminated PSCs under maximum power point tracking at Helmholtz-Zentrum Berlin (Germany). PR, performance retention. Reprinted with permission from Reference 75. © 2024 Royal Society of Chemistry.^55^

One of the first outdoor stability assessments of PSCs under outdoor conditions was carried out in Barcelona (Spain) by our group back in 2016. The MAPI-based PSCs sustained almost 1000 h under ISOS-3 protocol.^74^ Currently, excellent work is being carried out by Helmholtz-Zentrum Berlin with respect to PSC stability under outdoor conditions. Khenkin et al. have recently demonstrated the stability of PSCs under ISOS-O protocol for more than 22,000 h (2.5 years). Results of their work is depicted in **Figure** **2**. The devices are inverted PSCs of the type ITO/2PACz(SAM)/Cs_0.15_FA_0.85_PbI_0.55_Br_0.45_/C_60_/SnO_2_/Cu. In this case, the SAM molecules with phosphonate functional groups (MeO-2PACz and 2PACz) are present together with the adjacent oxide layer (ITO) next to the SAM molecule.^55^

**Pb-free perovskites.** Toxicity concerns on Pb-containing PSCs have delayed the widespread application of halide perovskite materials and has raised concerns about the commercialization of this PV technology.^8^ Berry et al. reminded us that electricity generation from coal results in more lead emitted into the atmosphere than PSCs (assuming a 20% module efficiency and an ∼700-nm active layer). In addition, some Pb-containing salts employed in PSCs are not water-soluble and thus, cannot be washed away by rain on a cracked panel. The rapid hydrolysis observed on Pb^2^^+^ materials results in the formation of Pb(OH)_2_, which is insoluble.^75^ The concern of the solubility of Pb(OH)_2_ and PbO in acid (acid rain) results in their transformation in to the corresponding sulfate species in pH equilibrium, also insoluble. Visoly-Fisher et al. also report the slow dissolution of Pb salts in water, which allows inspection and correction. In addition, Pb is used in paint and cosmetics and that might be a better starting point for prioritizing Pb removal.^76^

Despite all the latter, ongoing research to develop Pb-free halide perovskites has flourished in the past years. In halide perovskite materials, Pb can be replaced with other less-toxic, environmentally benign metals, including Sn, Ge, Bi, or Sb, among others. However, only Sn and Ge can form the perovskite crystal structure (they both have the same coordination, ion size, and charge balance).^77^ Sn is possibly the most promising alternative due to the similar semiconductor characteristics in comparison to Pb.^77^ This comes from the presence of the 6*s*2 (5*s*2) lone-pair electrons on the *B*-site only observed for Pb and Sn metals, which give these compounds their interesting properties. In addition, Sn is a low-toxicity element and Sn^2^^+^ degrades to eco-friendly SnO_2_ after exposure to air.^77^

**Figure 3:**
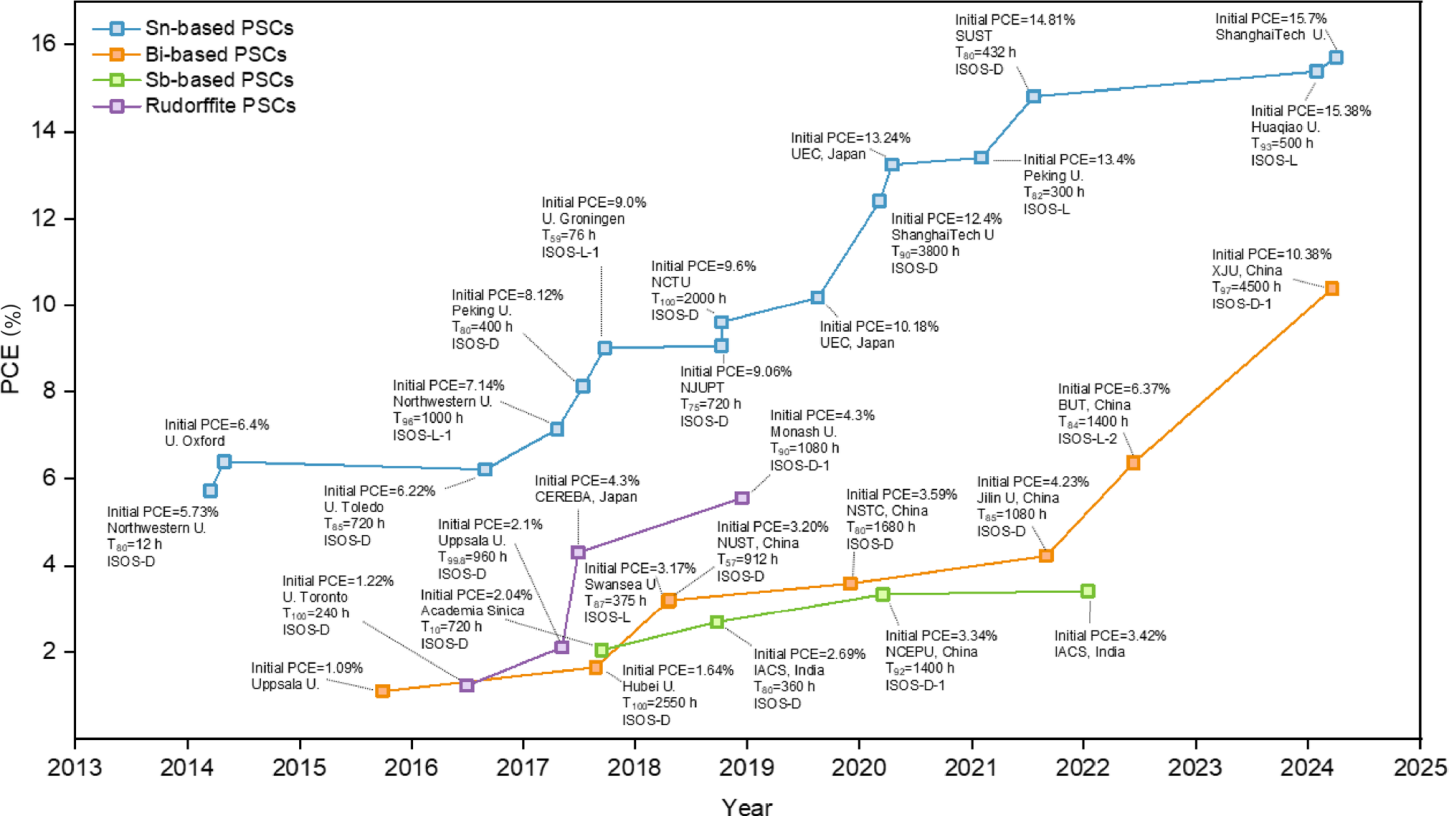
Timeline for the efficiency and the stability of some Pb-free perovskite solar cells (PSCs).  Sn-based;  Bi-based;  Sb-based;  Rudorfitte. For detailed data, please see Table SI in the Supplementary information. PCE, power-conversion efficiency.

**Figure 4:**
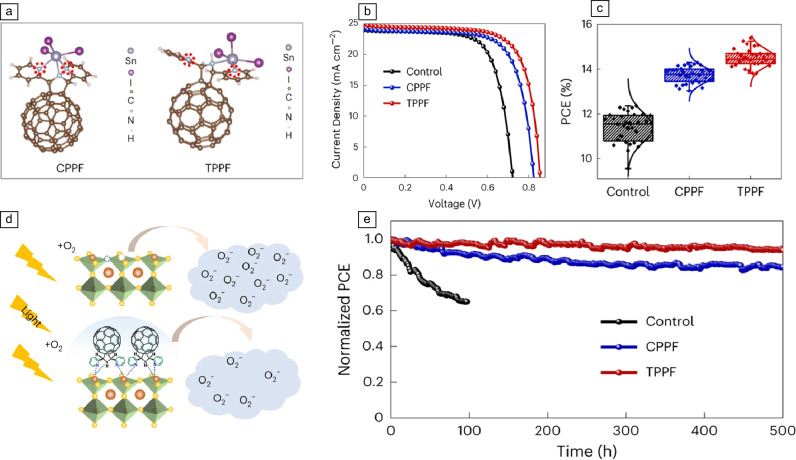
Efficiency and stability assessment of Pb-free perovskite solar cells of the type ITO/PEDOT:PSS/FASnI_3_(TPPF)/PCBM/BCP/Ag. (a) Molecular structure of the TPPF and CPPF, (b) *J*–*V* curves of the best-performing tin-based perovskite solar cells (TPSCs), (c) statistics of the power-conversion efficiency (PCE), (d) schematic illustration of the oxidation of DPBF by O_2_^−^ under different conditions, and (e) PCE evolution of the unencapsulated TPSCs under continuous illumination.^79^ Reprinted with permission from Reference 81. Springer Nature.

**Figure** **3** depicts the timeline for the efficiency and stability evolution of Pb-free halide perovskites for metals, such as Sn, Bi, Sb, and AgBi-based rudorffite perovskites (updated September 2024). The best conversion efficiency corresponds to Sn-based PSCs of the type ITO/PEDOT:PSS/FASnI_3_/FPEABr/ICBA/Ag with a record efficiency of 15.7% developed by ShanghaiTech University (China).^78^ In their solar cell they employed FASnI_3_ halide perovskite and introduced the molecule 4-fluorophenethylamine hydrobromide as an interfacial dipole between the perovskite and the electron-transport layers. The latter permitted to optimize the energy-level alignment between the layers, obtaining an open-circuit voltage of 0.974 V and 15.7% efficiency. However, no report on stability properties was made. Chen et al. from Huaqiao University (China), reported a Pb-free PSC of the type ITO/PEDOT:PSS/FASnI_3_(TPPF)/PCBM/BCP/Ag, with PCE of 15.38 percent. The authors employed two precursor additives from pyridyl-substituted fulleropyrrolidines (PPF) with the *cis* (CPPF) and *trans *(TPPF) configurations. These additives are meant to slow down the crystallization process affecting the electron density distribution and the interaction with the halide perovskite. Among both, the TPPF-based PSCs showed the highest efficiency of 15.38% (certified 15.14%) with a reported stability of *T*_99_ = 3000 h for ISOS-D, *T*_93_ = 500 h for ISOS-L (both in N_2_), *T*_98_ = 33.3 h for ISOS-D in air, and *T*_98_ = 11 h for ISOS-D in N_2_ and 85°C (see** Figure** **4**). It is reported that the TPPF additive can improve the interface energy-level alignment and suppress Sn^2^^+^ oxidation.^79^ These efficiency and stability data values for Pb-free PSCs, although limited, show the future opportunities for the application of Pb-free halide perovskites in PSCs.

**Figure 5:**
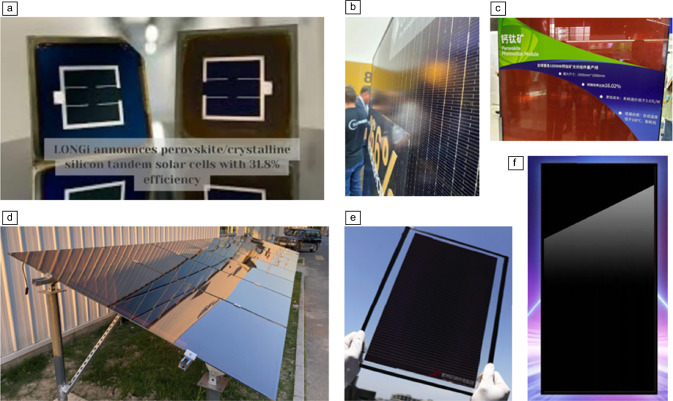
Images of some commercial examples of perovskite solar cells. (a) LONGi, (b) Oxford PV, (c) GCL-SI, (d) GCL-Si Installation, (e) Microquanta, (f) RenShine Solar PV.^84,87–91^

**Industry.** We are witnessing an important increase in the number of companies engaged in the fabrication and/or commercialization of PSCs (see** Figure** **5**). This growing interest is aligned with the predictions made for the global perovskite solar-cell market, which has been projected to grow at a constant rate in the next years, and to be situated between USD$2,000–4,000 million by ~2030.^80–82^ In 2023, major companies, many of them spinoff companies from well-recognized research laboratories, announced the commercialization of single-junction PSM modules with efficiencies of around 16%, which is a threshold mark if compared to commercialized silicon PV technology. Toshiba has claimed 16.6% efficiency of their single-junction PSM.^83^ GCL (China) reported their perovskite and perovskite-silicon tandem solar modules with efficiencies above 19% and 26%, respectively.^84^ RenShine Solar reports 18–20% efficiency for single-junction PSM and about 21% for tandem devices.^85^ Oxford PV has announced the commercialization of its tandem perovskite/Si modules with 24.5% efficiency, which can generate 20% more efficiency than silicon modules.^86^

**Figure 6:**
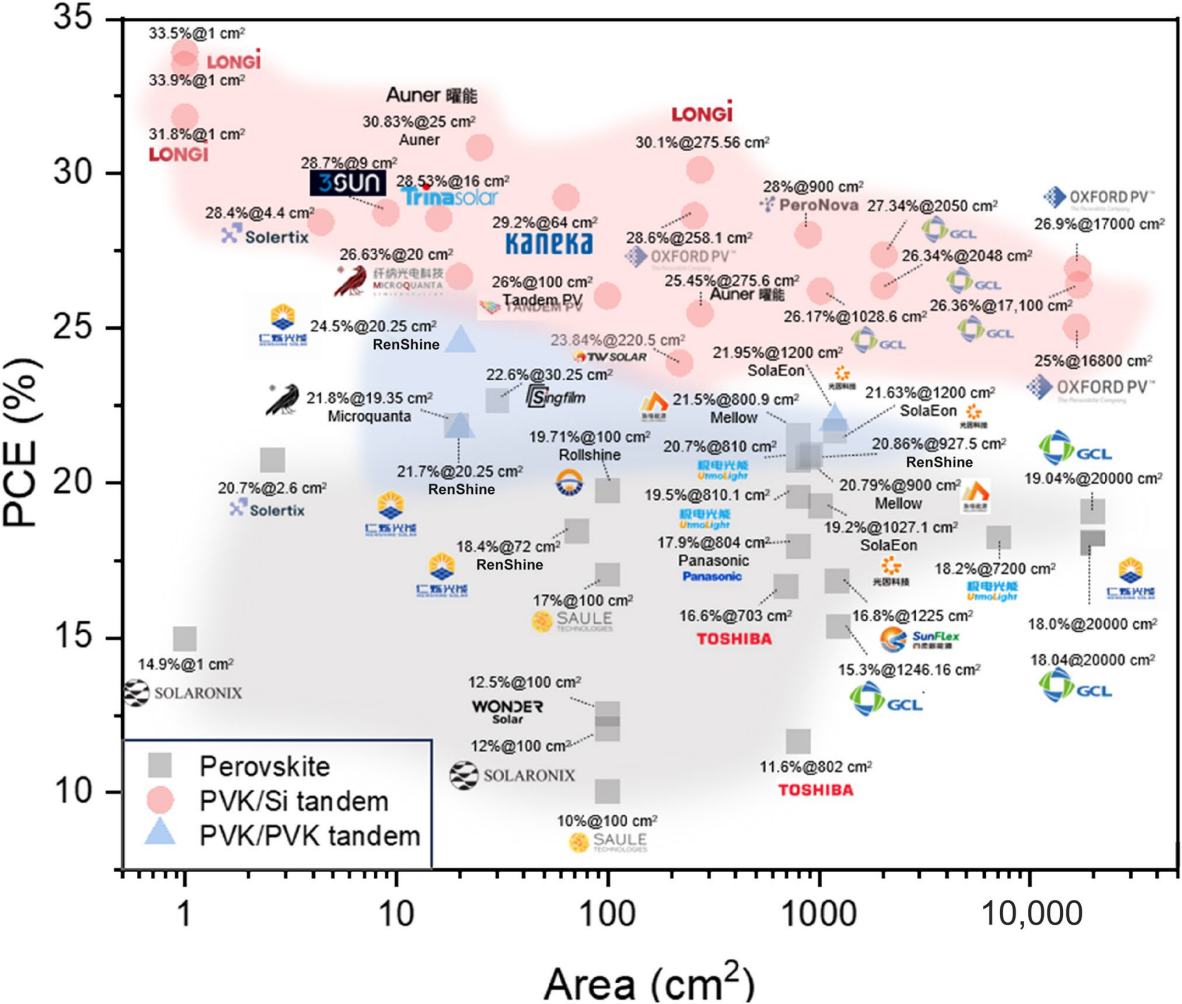
Power-conversion efficiency (PCE) for halide perovskite solar-cell perovskite solar modules developed by industries: single-junction (), perovskite/silicon tandem (), and perovskite/perovskite tandem () solar cells. For detailed information, please see Table SII in the Supplementary information. The graph encompasses certified and noncertified data.

Major PSC industry players are depicted in **Figure** **6** for single-junction and tandem modules. The graph depicts the PCE with respect to the active area (single cells) or aperture area (modules) and is separated by the type of PSC architecture: single-junction (), perovskite/silicon tandem (), and perovskite/perovskite tandem () solar cells. It has been already reported that the final PCE of laboratory-scale single-cell PSCs depends on the devices active (with small active areas usually showing the largest PCE).^92^ However, we can observe in Figure 6 that the situation seems not to be the same for industrial single and tandem modules. PCEs for single-junction modules are reported to be between 19% and 21.5% for a large range of active areas that varies between 800 cm^2^ and 20,000 cm^2^ (here we are not considering the difference made between laboratory-scale devices and industrial modules). Some differences can be observed within a single company; for example, RenShine reports efficiencies between 18% and 21% for active areas between 25 cm^2 ^and 100 cm^2^; SolaEon reports PCEs from 19% to 21% for active areas between 1000 cm^2^ and 1200 cm^2^ for single modules and between 21% and 29% for tandem modules with area of 300 mm × 400 mm. Compared with single-junction cells, all-perovskite tandem cells require accurate control of the different layers of the tandem device. This involves careful optimization of materials formulation and thin-film deposition thickness and homogeneity, including material stability and preparation process. In comparison of the PCE for PVSK/Si tandem modules, we can find PCEs between 30% and 33.9% for modules with varying active area from 1 cm^2^ up to 25.832 cm^2^ (LONGi), or between 26% and 27% PCE for areas between 1 cm^2^ and 17,000 cm^2^ (GCL). Oxford PV reported 25–28.6% for active areas between 1 cm^2^ and 16,800 cm^2^. SolaEon, a Chinese manufacturer, has reported a 29.34% PCE for monolithic PVSK/SI tandem solar cells and 21.95% for a 300 mm × 400 mm monolithic tandem PSC.

It is important to note that data shown in Figure 6, and the corresponding Table SII, represent values obtained from published sources and thus, their variability can be substantial. PCE values coming from the same device/module can differ greatly if coming from certified or noncertified sources, if they represent the maximum or the steady-state efficiency value or if these are obtained in reverse or forward scans. Especially certified values obtained by recognized laboratories usually result in lower PCEs.

Although many companies are not disclosing their stability analysis results and PSC lifetime, a few have done so, especially for single-cell modules, with very promising outcomes. For single- cell devices, Utmo Light (China) reported the positive result of their PSM passing the IEC testing, which can withstand a 2300-h UV bath at 1000 W/m^2^ and 60°C, and predicted 12 years of operation without degradation.^93^ GCL’s perovskite solar panel had passed the IEC 61215 and IEC 61739 certification tests employed for silicon solar cells. The company guarantees 90% of the nominal output power after 10 years, which can decrease to 80% after 25 years.^84^ Rayleigh Solar Tech has announced zero degradation for their 15 cm × 15 cm glass solar module after seven months (>5.000 h) of outdoor testing and T_80_ of 1200 h for their flexible module of the same size under damp heat test.^94^

RenShine solar, a spinoff company led by T. Hairen from Nanjing University, China, reported that their PSCs are capable of retaining greater than 90% of their initial performance after 600 h of continuous operation.^95^ Their company has finalized the construction of a 1.2 MW perovskite rooftop distributed power station, which was connected to the grid on September 2024. It encompasses 9.300 commercial PSM (1.2 m × 0.6 m, average power of 130 W). It has reported that its commercial modules have achieved a power-conversion efficiency of 18.4 percent. RenShine Solar has signed an agreement for a CNY 1 billion investment in PSCs.^90^

**Figure 7:**
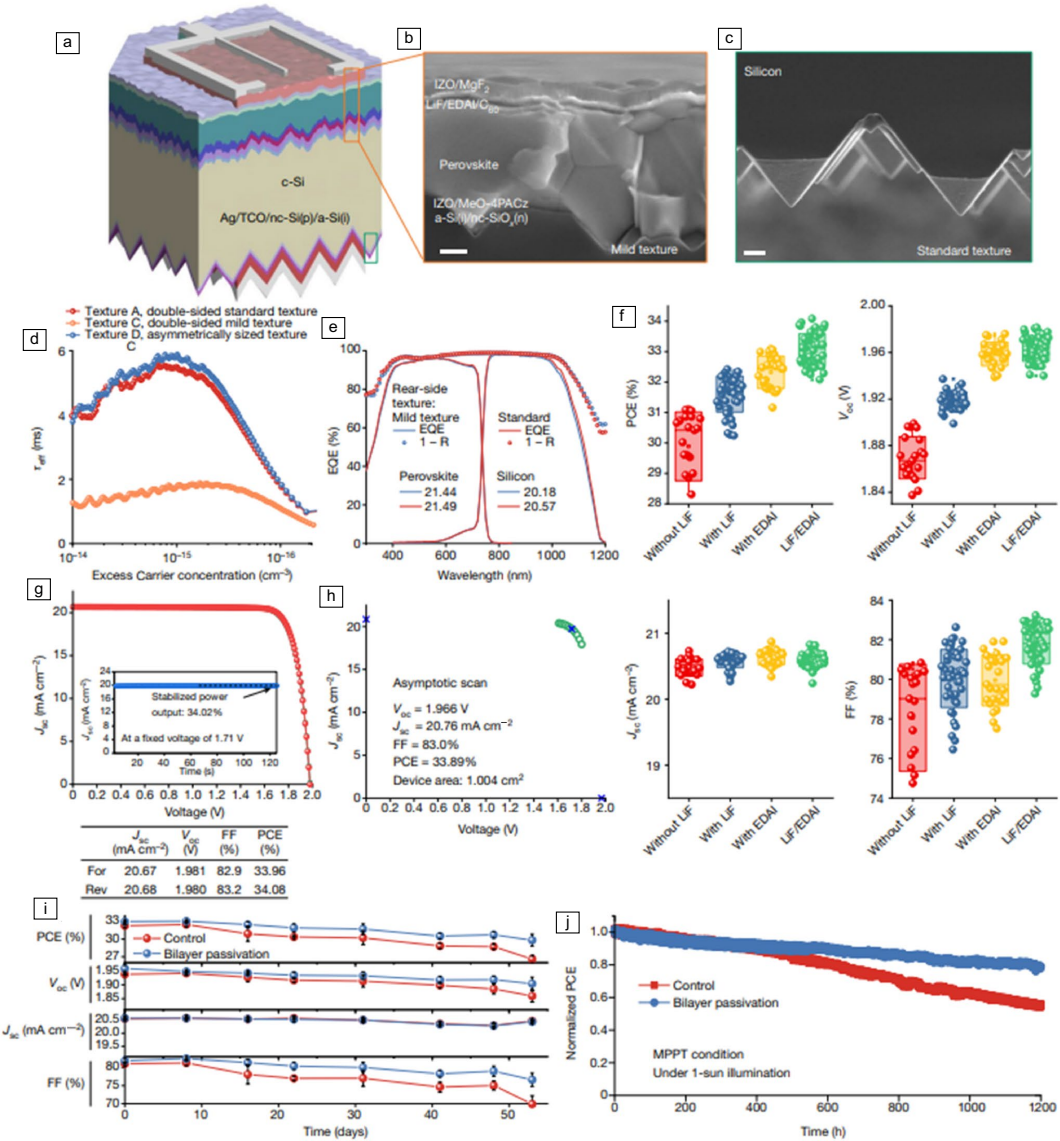
Tandem silicon/perovskite solar cells applying the LiF/EDAI bilayer. (a) Schematic of the monolithic perovskite/silicon tandem solar cell, (b) and standard-sized pyramid texture on the rear side (c). Scale bars = 200 nm (b) and 1 μm (c). (d) Effective minority carrier lifetime (τ_eff_) measurements. (e) External quantum efficiency (EQE) comparison of the perovskite/silicon tandems using mild texture and standard texture on the rear side. (f) Photovoltaic performance parameter statistics for 1 cm^2^ perovskite/silicon tandem. (g) In-house* J–V* curves. PCE, power-conversion efficiency. (h) * J–V* curve and the maximum power output point of one tandem cell, measured by the National Renewable Energy Laboratory. (i) Stability under ISOS-D protocol for the control (LiF treated) and bilayer passivated tandems. (j) Stability under ISOS-L protocol at room temperature.^1^ MPPT, maximum power point tracking, FF, fill factor. Reprinted with permission from Reference 1.  © 2024 Springer Nature.

In 2024, tandem silicon/perovskite solar cells exceeded, for the first time, the single-junction Shockley–Queisser limit of 33.7 percent. The work was developed through an academy/industry collaboration and involving LONGi Green Energy Technology Co. in collaboration with Soochow University and eight other institutions in China and Hong Kong (**Figure** **7**). These two-terminal monolithic perovskite/silicon tandem solar cells were produced by a holistic approach^96^ with a bilayer-intertwined passivation. The authors applied a lithium fluoride ultrathin layer followed by an additional deposition of diammonium diiodide molecule (LiF/EDAI bilayer) enhancing electron charge extraction and suppressing nonradiative recombination. As a result, they obtained an independently certified stabilized PCE of 33.89% with an excellent FF of 83% and 1.97 *V*_oc_.^1^ The reported stability for their PSC applying the LiF/EDAI bilayer was* T*_80_ of 1200 h obtained under ISOS-L conditions of MPP tracking, continuous illumination under N_2_ (Figure 7j). The later result emphasizes the importance of employing a holistic approach to obtain highly efficient devices.^96^

**Figure 8:**
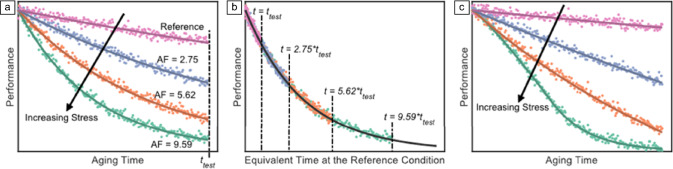
Prediction of long-term solar-cell performance degradation using accelerated aging tests. (a) Computer-generated pseudo data comparing solar-cell performance evolution under reference and accelerated aging conditions for a test duration, test. Degradation rates for each curve were used to calculate their acceleration factor (AF) as indicated next to each curve. (b) The data of (a) are plotted on a unified “equivalent time at the reference condition” = ttest.AF. (c) Pseudo data demonstrating a case where the functional form of the degradation curves at elevated stress conditions is different from the reference, and thus AFs cannot be calculated. Reprinted with permission from Reference 97. © 2023 Springer Nature. Reprinted with permission from Reference 98. © 2011 Elsevier.

**Accelerated aging.** Accelerated aging testing, while technically challenging, is the key to rapid assessment of the operational lifetimes and elucidation of long-term degradation mechanisms. If effectively applied, it can help the technology to be derisked and its time to market can be expedited. However, could accelerated tests properly predict the real-world operational lifetimes or at least that measured with aging under the STCs?^97^ If the PCE versus time curves exhibit a consistent mathematical form (e.g., linear, exponential decay) with increasing stress (for the intrinsic degradation, it can be heating or light exposure), the rate of existing degradation processes is likely being accelerated (**Figure** **8**a–b). In contrast, a notable change in the degradation curve shape indicates that new degradation modes are likely being activated (Figure 8c).

For data like those in Figure 8a, that exhibit a consistent functional form with increasing stress, an acceleration factor (AF) can be defined for each stress level by dividing the degradation rate of solar cells aging at each accelerated condition by the rate of those aging at the reference condition (STC or outdoor). In the case of Figure 8a, the data from different stress conditions can be plotted onto a unified “equivalent time at the reference condition” axis as shown in Figure 8b, where all data collapse onto a single unified curve—the hallmark of an effective accelerated aging test. Because the data in Figure 8c do not have the same shape, acceleration factors cannot be extracted.

As previously stated, internal degradation mechanisms for PSCs can be thermally activated and/or photo-induced. This can be expressed as:^98^

1 $$\frac{{k_{{{\text{acc}}}} }}{{k_{{{\text{ref}}}} }} = \text{AF}\left( {T_{{{\text{acc}}}} ,I_{{{\text{acc}}}} } \right) = \left( {\frac{{I_{{{\text{acc}}}} }}{{I_{{{\text{ref}}}} }}} \right){\text{exp}}\left( {\frac{{E_{a} }}{{k_{\text{B}} }}\left( {\frac{1}{{T_{{{\text{ref}}}} }} - \frac{1}{{T_{{{\text{acc}}}} }}} \right)} \right),$$

where *E*_*a*_ is the activation energy of degradation,* k*_B_ is the Boltzmann constant,* T*_acc_,* I*_acc_, and* k*_acc_ are the temperature, light intensity, and the rate of solar-cell degradation at the accelerated condition, and* T*_ref_,* I*_ref_, and* k*_ref_ are the temperature, light intensity, and the degradation rate at the reference condition.

This approach can properly predict the real-world operational lifetimes preferably for the most stable devices for which one degradation mechanism dominates and the stress factor is known. Recently, it was successfully demonstrated for PSC^17^ and PSM^99^ with variation of temperatures in the ISOS-L aging as well as with aging under various intensities of concentrated sunlight.^100^ In the latter case, light intensity and the cell temperature should be controlled independently.^101^

## Conclusion

In summary, PSCs are just now entering the commercial stage. Enormous efforts are being dedicated to demonstrating the long-lifetime stability of laboratory-scale solar cells and commercial modules. Currently, outdoor stability analyses of laboratory-scale devices can prove above 20,000 h stability under ISOS-O protocol, and several thousand hours for ISOS-L under harsh temperatures of 85°C. Many commercial modules have passed the IEC standardization required for silicon solar cells, which gives a promising future for the technology. The answer to the question “Are we there yet?” will be solved in the next couple of years once this PV technology demonstrate its endurance and long lifespan.

## Supplementary Information

Below is the link to the electronic supplementary material.

Supplementary file1 (DOCX 59 kb)

## Data Availability

Not applicable.
