# Supplementary material for: Stability and reliability of perovskite photovoltaics: Are we there yet?
Source: MRS Bull. 2025 Mar 18;50(4):512–25. doi: 10.1557/s43577-025-00863-5 (PMC11985620; doi:10.1557/s43577-025-00863-5)
Supplement: Supplementary file 1 — Supplementary file1 (DOCX 59 kb) [file 43577_2025_863_MOESM1_ESM.docx]

Table S1. The timeline table for the efficiency and stability evolution of Pb-free Halide perovskites for metals (Sn, Bi, Sb and AgBi-based Rudoffite perovskites).

| **Tin-based Perovskite Solar Cells** | | | | | | |
| --- | --- | --- | --- | --- | --- | --- |
| **Year** | **Structure** | **Initial**  **PCE** | **Inst.** | **Life time** | **Test condition** | **Ref** |
| 2014.3.18 | FTO/TiO2/CH3NH3SnI3-XBrX/Spiro-OMeTAD/Au | 5.73 | Northwestern U | T80=12 h | dark storing in N2, JV test regularly | ^1^ |
| 2014.5.1 | FTO/TiO2/CH3NH3SnI3/Spiro-OMeTAD/ | 6.4 | U Oxford | none | none | ^2^ |
| 2016.8.29 | ITO/PEDOT:PSS/FASnI3/PCBM/BCP/Ag | 6.22 | U Toledo | T85=30 d | dark storing in N2, JV test regularly | ^3^ |
| 2017.4.21 | FTO/TiO2/FASnI3/PTAA/Au | 7.14 | Northwestern U | T80=40 min | AM 1.5G, ambient air (ISOS-L-1) | ^4^ |
|  |  |  |  | T96=1000 h | dark storing in N2 |  |
| 2017.7.14 | ITO/PEDOT:PSS/ FA0.75MA0.25SnI3/PCBM/BCP/Ag | 8.12 | Peking U | T80=400 h | storing in N2, JV test regularly | ^5^ |
| 2017.9.22 | ITO/PEDOT:PSS/FASnI3/PCBM/BCP/Al | 9.0 | U.Groningen | T96≈2 h | continuous 1 sun light in N2 | ^6^ |
|  |  |  |  | T59=76 h | continuous 1 sun light in 20% humidity |  |
| 2018.10.9 | ITO/PEDOT:PSS/ FA0.75MA0.25SnI3/PCBM/BCP/Al | 9.06 | NJUPT | T75=30 d | dark storing in N2, JV test regularly | ^7^ |
| 2018.10.9 | ITO/PEDOT:PSS/GAxFA(0.98−x)SnI3–1%EDAI2/PCBM/BCP/Ag | 9.6 | NCTU, Taiwan | T80=96 h, T30=150 h | Dark storing in RH 60%, JV test regularly | ^8^ |
|  |  |  |  | T100=170 h | Dark storing in RH 20%, JV test regularly |  |
|  |  |  |  | T100=2000 h | Dark storing in N2, JV test regularly  PCE increased from 7.3% to 9.6%. |  |
| 2019.8.18 | FTO/PEDOT:PSS/FA0.98EDA0.01SnI3/ PCBM/BCP/Au Ag | 10.18 | UEC, Japan | none | none | ^9^ |
| 2020.3.6 | ITO/PEDOT/PEA0.15FA0.85SnI3-SCN/ICBA/BCP/Ag | 12.4 | ShanghaiTech U | T90=3800 h | dark storing in N2, JV test regularly | ^10^ |
|  |  |  |  | T50=5 h | AM 1.5G, OC in N2 ISOS-L |  |
| 2020.4.16 | FTO/PEDOT:PSS/ GeI2-(FA0.9EA0.1)0.98EDA0.01SnI3/PCBM/BCP/Ag/Au | 13.24 | UEC, Japan | none | none | ^11^ |
| 2021.2.3 | ITO/PEDOT:PSS/FASnI3/PCBM/BCP/Ag | 13.4 | Peking U | T91=4800 h | dark storing in N2, JV test regularly | ^12^ |
|  |  |  |  | T82=300 h | AM 1.5G, OC in N2, ISOS-L |  |
| 2021.7.23 | ITO/PEDOT:PSS/FASnI3/FPEABr(2D/3D)/PCBM/BCP/Al | 14.81 | SUST, China | T80=432 h | dark storing in N2, JV test regularly | ^13^ |
|  |  |  |  | T30≈0.5 h | dark storing in Air RH 75%, JV test regularly |  |
|  |  |  |  | T65≈ 1h | Heating press, 85 °C, JV test regularly |  |
|  |  |  |  | T70≈ 2.5 h | AM 1.5G, JV test regularly |  |
| 2024.1.29 | ITO/PEDOT:PSS/FASnI3(TPPF)/PCBM/BCP/Ag | 15.38 | Huaqiao U, China | T99=3000 h | dark storing in N2, JV test regularly | ^14^ |
|  |  |  |  | T93=500 h | AM 1.5G in N2?, MPP? |  |
|  |  |  |  | T98= 33.3 h | dark storing in Air RH 30%, JV test regularly |  |
|  |  |  |  | T100=11 h | dark storing in N2, heating 85 °C, JV test regularly |  |
| 2024.4.2 | ITO/PEDOT:PSS/FASnI3/FPEABr/ICBA/Ag | 15.7 |  | None | None | ^15^ |
| **Bismuth-based Perovskite Solar Cells** | | | | | | |
| **Year** | **Structure** | **Initial**  **PCE** | **Inst.** | **Life time** | **Test condition** | **Ref** |
| 2015.9.29 | FTO/TiO2/Cs3Bi2I9/HTL/Au | 1.09 | Uppsala U | none | dark storing in air RH 10%, JV test regularly, PCE from 0.17 to 0.29. | ^16^ |
| 2017.8.25 | FTO/TiO2/ MA3Bi2I9/ Spiro-OMeTAD/Au | 1.64 | Hubei U | T100=2550 h | dark storing in N2, JV test regularly | ^17^ |
| 2018.4.16 | FTO/TiO2/MA3Bi2I9/P3HT/Au | 3.17 | Swansea U | T97=1440 h | ?dark storing in air, JV test regularly? | ^18^ |
|  |  |  |  | T87=375 h | AM 1.5G in air, MPPT, ISOS-L-1 |  |
| 2018.4.21 | FTO/TiO2/Cs3Bi2I9/CuI | 3.20 | NUST, China | T57=912 h | dark storing in air RH 45%, JV test regularly | ^19^ |
| 2019.12.2 | FTO/TiO2/0.5Cs3Bi2I9-Ag3Bi2I9/PDBD-T/Au | 3.59 | USTC, China | T80=1680 h | dark storing in air RH 35%, JV test regularly | ^20^ |
|  |  |  |  | T90=450 h | dark storing in N2, and heating at 85 degrees |  |
| 2021.9.1 | FTO/TiO2/Cs2AgBiBr6/Spiro-OMeTAD/Ag | 4.23 | Jilin U | T85=1080 h | dark storing in air, JV test regularly | ^21^ |
|  |  |  |  | T98=6 h | AM 1.5G in air, MPPT |  |
| 2022.6.13 | ITO/SnO2/Cs2AgBiBr6/Spiro-OMeTAD/Au | 6.37 | BUT, China | T95=1400 h | Light illumination in N2 at 20 degrees | ^22^ |
|  |  |  |  | T91=1400 h | Dark storing in N2 at 85 degrees, JV test regularly |  |
|  |  |  |  | T84=1400 h | Light illumination in N2 at 85 degrees |  |
|  |  |  |  | T42=1400 h | Light illumination in air RH 85% at 85 degrees |  |
| 2024.3.19 | FTO/TiO2/CsBiSCl2/Spiro-OMeTAD/Au | 10.38 | SJTU, China | T97=4500 h | Dark storing in air, JV test regularly | ^23^ |
|  |  |  |  | T93=200 h | AM 1.5G, light illumination |  |
| **Sb-based Perovskite Solar Cells** | | | | | | |
| **Year** | **Structure** | **Initial**  **PCE** | **Inst.** | **Life time** | **Test condition** | **Ref** |
| 2017.9.13 | ITO/PEDOT:PSS/MA3Sb2I9/PCBM/BCP/Al | 2.04 | Academia Sinica, Taiwan | T10=720 h | Dark storing in N2, JV test regularly | ^24^ |
| 2018.9.25 | ITO/Cu:NiO/MA3Sb2I9/ZnO/Al | 2.69 | IACS, India | T80=360 h | Dark storing in air, JV test regularly | ^25^ |
| 2020.3.16 | FTO/TiO2/MA3Sb2I9–*x*Cl*x*/Spiro-OMeTAD/Au | 3.34 | NCEPU, China | T90=4000 h | Room light illumination in N2, OC | ^26^ |
|  |  |  |  | T92=1400 h | Dark storing in air RH 30%, JV test regularly |  |
| 2022.1.15 | ITO/PEDOT:PSS/Cs3Sb2ClxI9−x/PCBM/Al | 3.42 | IACS, India | none | none | ^27^ |
| **Rudorffite AgBiX solar cells** | | | | | | |
| **Year** | **Structure** | **Initial**  **PCE** | **Inst.** | **Life time** | **Test condition** | **Ref** |
| 2016.6.29 | FTO/TiO2/AgBi2I7/P3HT/Au | 1.22 | U Toronto | T100=240 h | Dark storing in air, JV test regularly | ^28^ |
| 2017.5.8 | FTO/TiO2/Ag2BiI5/P3HT/Au | 2.10 | Uppsala U | T99.8=960 h | Dark storing in N2, JV test regularly | ^29^ |
| 2017.6.28 | FTO/TiO2/Ag3BiI6/PTAA/Au | 4.30 | CEREBA, Japan | none | none | ^30^ |

Table S2. Single cell modules and Tandem modules from industry. The table encompasses certified and no-certified data.

| **Company** | **Module Eff (%)**  **(c=certified)** | **Aperture/Active area**  **(cm^2^)** | **Ref** |
| --- | --- | --- | --- |
| **Single cell modules** | | | |
| Microquanta Semiconductor | 21.8 | 19.35 | ^31, 32^ |
| Wuxi Utmolight Technology | 19.5 | 810.1 | ^33^ |
|  | 18.2 | 7200 | ^34^ |
|  | 20.7 | 810 | ^35^ |
| Kunshan GCL Optoelectronic Material | 15.31 | 1246.16 | ^36^ |
|  | 18.04 | 20000 |  |
|  | 19.04 | 20000 |  |
| WonderSolar | 12.5 | 100 | ^37^ |
| Toshiba | 11.6 | 802 | ^38-40^ |
|  | 16.6 | 703 |  |
| Panasonic | 17.9 | 804 | ^41^ |
| Saule Technologies | 17  10 | 100 | ^42, 43^ |
| Solaronix SA | 14.9  12 | 1  100 | ^42, 44^ |
| Renshine Solar | 18 | 20000 | ^45^ |
|  | 18.4 | 72 |  |
|  | 20.86 | 927.5 |  |
| DaZheng | 13-15 | 6000 |  |
| SolaEon Technology | 19.2 c | 1027.1 | ^46^ |
|  | 21.63 | 1200 |  |
| Mellow Energy | 21.5 | 800.9 | ^47^ |
|  | 20.79 | 900 |  |
| Singfilm Solar | 22.6 | 30.25 | ^48^ |
| [Solertix](https://www.pv-magazine.com/2023/06/01/futurasun-acquires-solar-perovskite-startup/) | 20.7 | 2.6 | ^49^ |
| Sunflex | 16.8 | 1225 | ^50^ |
| Rollshine | 19.71 | 100 | ^51^ |
| Solliance | 12.6 | 0.1 | ^52^ |
| **PSC/Si Tandem** | | | |
| LONGI | 33.9 | 1 | ^53^ |
|  | 33.5 | 1 | ^54^ |
|  | 31.8 | 1 | ^55^ |
|  | 30.1 | 275.56 | ^56^ |
| Auner | 25.45  30.83 | 275.6  25 | ^45^ |
| Tongwei | 23.84 | 220.5 |  |
| KANEKA | 29.20 | 64 | ^45^ |
| OXFORD PV | 28.60 | 258.1 | ^57^ |
|  | 25.0 | 16800 (1.68 m^2^) | ^58^ |
|  | 26.9 | 17000 (~1.6 m^2^, 1m x 1.7m) | ^59^ |
| Trina Solar | 28.53 | 16 | ^60^ |
| GCL | 27.34 | 2,050 | ^45^ |
|  | 26.36 | 17,100 |  |
|  | 26.34 | 2,048 |  |
|  | 26.17 | 1028.6 |  |
| Tandem PV | 26 | 100 | ^61^ |
| PeroNova | 28 | 900 | ^62^ |
| Microquanta Semiconductor | 26.63 | 20 | ^63^ |
| Solertix | 28.4 | 4.4 | ^64^ |
| 3Sun | 28.7 | 9 | ^65^ |
| **PSC/PSC Tandem** | | | |
| Renshine Solar | 24.50 | 20.25 | ^66^ |
|  | 21.7 | 20.25 |  |
| SolaEon Technology | 21.95 | 1200 | ^46^ |

**References**

1. F. Hao, C.C. Stoumpos, D.H. Cao, R.P.H. Chang and M.G. Kanatzidis, *Nat. Photonics.* **8**, 489-494 (2014).<https://doi.org/10.1038/nphoton.2014.82>

2. N.K. Noel, S.D. Stranks, A. Abate, C. Wehrenfennig, S. Guarnera, A.-A. Haghighirad, A. Sadhanala, G.E. Eperon, S.K. Pathak, M.B. Johnston, A. Petrozza, L.M. Herz and H.J. Snaith, *Energy Environ. Sci.* **7**, 3061-3068 (2014).<https://doi.org/10.1039/C4EE01076K>

3. W. Liao, D. Zhao, Y. Yu, C.R. Grice, C. Wang, A.J. Cimaroli, P. Schulz, W. Meng, K. Zhu, R.G. Xiong and Y. Yan, *Adv. Mater.* **28**, 9333-9340 (2016).<https://doi.org/10.1002/adma.201602992>

4. W. Ke, C.C. Stoumpos, M. Zhu, L. Mao, I. Spanopoulos, J. Liu, O.Y. Kontsevoi, M. Chen, D. Sarma and Y. Zhang, *Science advances.* **3**, e1701293 (2017).<https://doi.org/10.1126/sciadv.1701293>

5. Z. Zhao, F. Gu, Y. Li, W. Sun, S. Ye, H. Rao, Z. Liu, Z. Bian and C. Huang, *Advanced Science.* **4**, 1700204 (2017).<https://doi.org/10.1002/advs.201700204>s

6. S. Shao, J. Liu, G. Portale, H.-H. Fang, G.R. Blake, G.H. ten Brink, L.J.A. Koster and M.A. Loi, *Advanced Energy Materials.* **8**, 1702019 (2018).<https://doi.org/10.1002/aenm.201702019>

7. X. Liu, K. Yan, D. Tan, X. Liang, H. Zhang and W. Huang, *ACS Energy Lett.* **3**, 2701-2707 (2018).<https://doi.org/10.1021/acsenergylett.8b01588>

8. E. Jokar, C.H. Chien, C.M. Tsai, A. Fathi and E.W.G. Diau, *Adv. Mater.* **31**, 1804835 (2019).<https://doi.org/10.1002/adma.201804835>

9. M.A. Kamarudin, D. Hirotani, Z. Wang, K. Hamada, K. Nishimura, Q. Shen, T. Toyoda, S. Iikubo, T. Minemoto, K. Yoshino and S. Hayase, *The Journal of Physical Chemistry Letters.* **10**, 5277-5283 (2019).<https://doi.org/10.1021/acs.jpclett.9b02024>

10. X. Jiang, F. Wang, Q. Wei, H. Li, Y. Shang, W. Zhou, C. Wang, P. Cheng, Q. Chen, L. Chen and Z. Ning, *Nat. Commun.* **11**, 1245 (2020).<https://doi.org/10.1038/s41467-020-15078-2>

11. K. Nishimura, M.A. Kamarudin, D. Hirotani, K. Hamada, Q. Shen, S. Iikubo, T. Minemoto, K. Yoshino and S. Hayase, *Nano Energy.* **74**, 104858 (2020).<https://doi.org/10.1016/j.nanoen.2020.104858>

12. C. Wang, Y. Zhang, F. Gu, Z. Zhao, H. Li, H. Jiang, Z. Bian and Z. Liu, *Matter.* **4**, 709-721 (2021).<https://doi.org/10.1016/j.matt.2020.11.012>

13. B.-B. Yu, Z. Chen, Y. Zhu, Y. Wang, B. Han, G. Chen, X. Zhang, Z. Du and Z. He, *Adv. Mater.* **33**, 2102055 (2021).<https://doi.org/10.1002/adma.202102055>

14. J. Chen, J. Luo, E. Hou, P. Song, Y. Li, C. Sun, W. Feng, S. Cheng, H. Zhang, L. Xie, C. Tian and Z. Wei, *Nat. Photonics.* **18**, 464-470 (2024).<https://doi.org/10.1038/s41566-024-01381-7>

15. Y. Shi, Z. Zhu, D. Miao, Y. Ding and Q. Mi, *ACS Energy Lett.* **9**, 1895-1897 (2024).<https://doi.org/10.1021/acsenergylett.4c00529>

16. B.-W. Park, B. Philippe, X. Zhang, H. Rensmo, G. Boschloo and E.M.J. Johansson, *Adv. Mater.* **27**, 6806-6813 (2015).<https://doi.org/10.1002/adma.201501978>

17. Z. Zhang, X. Li, X. Xia, Z. Wang, Z. Huang, B. Lei and Y. Gao, *The Journal of Physical Chemistry Letters.* **8**, 4300-4307 (2017).<https://doi.org/10.1016/j.matt.2020.11.012>

18. S.M. Jain, D. Phuyal, M.L. Davies, M. Li, B. Philippe, C. De Castro, Z. Qiu, J. Kim, T. Watson, W.C. Tsoi, O. Karis, H. Rensmo, G. Boschloo, T. Edvinsson and J.R. Durrant, *Nano Energy.* **49**, 614-624 (2018).<https://doi.org/10.1016/j.nanoen.2018.05.003>

19. F. Bai, Y. Hu, Y. Hu, T. Qiu, X. Miao and S. Zhang, *Sol. Energy Mater. Sol. Cells.* **184**, 15-21 (2018).<https://doi.org/10.1016/j.solmat.2018.04.032>

20. W. Hu, X. He, Z. Fang, W. Lian, Y. Shang, X. Li, W. Zhou, M. Zhang, T. Chen, Y. Lu, L. Zhang, L. Ding and S. Yang, *Nano Energy.* **68**, 104362 (2020).<https://doi.org/10.1016/j.nanoen.2019.104362>

21. B. Wang, N. Li, L. Yang, C. Dall’Agnese, A.K. Jena, T. Miyasaka and X.-F. Wang, *Journal of the American Chemical Society.* **143**, 14877-14883 (2021).<https://doi.org/10.1021/jacs.1c07200>

22. Z. Zhang, Q. Sun, Y. Lu, F. Lu, X. Mu, S.-H. Wei and M. Sui, *Nat. Commun.* **13**, 3397 (2022).<https://doi.org/10.1038/s41467-022-31016-w>

23. J. Huang, H. Wang, C. Jia, H. Yang, Y. Tang, K. Gou, Y. Zhou and D. Zhang, *The Journal of Physical Chemistry Letters.* **15**, 3383-3389 (2024).<https://doi.org/10.1021/acs.jpclett.4c00310>

24. K.M. Boopathi, P. Karuppuswamy, A. Singh, C. Hanmandlu, L. Lin, S.A. Abbas, C.C. Chang, P.C. Wang, G. Li and C.W. Chu, *Journal of Materials Chemistry A.* **5**, 20843-20850 (2017).<http://dx.doi.org/10.1039/C7TA06679A>

25. S. Chatterjee and A.J. Pal, *ACS Appl. Mater. Interfaces.* **10**, 35194-35205 (2018).<https://doi.org/10.1021/acsami.8b12018>

26. Y. Yang, C. Liu, M. Cai, Y. Liao, Y. Ding, S. Ma, X. Liu, M. Guli, S. Dai and M.K. Nazeeruddin, *ACS Appl. Mater. Interfaces.* **12**, 17062-17069 (2020).<https://doi.org/10.1021/acsami.0c00681>

27. G. Paul and A.J. Pal, *Solar Energy.* **232**, 196-203 (2022).<https://doi.org/10.1016/j.solener.2021.12.056>

28. Y. Kim, Z. Yang, A. Jain, O. Voznyy, G.-H. Kim, M. Liu, L.N. Quan, F.P. García de Arquer, R. Comin, J.Z. Fan and E.H. Sargent, *Angew. Chem. Int. Ed.* **55**, 9586-9590 (2016).<https://doi.org/10.1002/anie.201603608>

29. H. Zhu, M. Pan, M.B. Johansson and E.M.J. Johansson, *ChemSusChem.* **10**, 2592-2596 (2017).<https://doi.org/10.1002/cssc.201700634>

30. I. Turkevych, S. Kazaoui, E. Ito, T. Urano, K. Yamada, H. Tomiyasu, H. Yamagishi, M. Kondo and S. Aramaki, *ChemSusChem.* **10**, 3754-3759 (2017).<https://doi.org/10.1002/cssc.201700980>

31. Microquanta, 21.8%, Microquanta won the champion in the perovskite field of CPVS Best Research-Cell Efficiencies, 2023, <https://www.microquanta.com/#/pc/new/125>.

32. P. Zhu, C. Chen, J. Dai, Y. Zhang, R. Mao, S. Chen, J. Huang and J. Zhu, *Adv. Mater.* **36**, 2307357 (2024).<https://doi.org/10.1002/adma.202307357>

33. M.A. Green, E.D. Dunlop, M. Yoshita, N. Kopidakis, K. Bothe, G. Siefer and X. Hao, *Progress in Photovoltaics: Research and Applications.* **32**, 3-13 (2024).<https://doi.org/10.1002/pip.3750>

34. R. Peleg, Wuxi UtmoLight Technology announces 18.2% efficiency for large area perovskite solar module, 2020, <https://www.perovskite-info.com/wuxi-utmolight-technology-announces-182-efficiency-large-area-perovskite-solar>.

35. R. Peleg, Wuxi UtmoLight reports 20.7% steady-state efficiency for its perovskite solar modules, 2024, <https://www.perovskite-info.com/wuxi-utmolight-reports-207-steady-state-efficiency-its-perovskite-solar-modules>.

36. PVTIME, 19.04%! GCL Perovskite Hits High Conversion Efficiency on Single-junction Perovskite Modules (1,000mmx2,000mm), 2024, <https://www.pvtime.org/19-04-gcl-perovskite-hits-high-conversion-efficiency-on-single-junction-perovskite-modules-1000mmx2000mm/>.

37. A. Mei, Y. Sheng, Y. Ming, Y. Hu, Y. Rong, W. Zhang, S. Luo, G. Na, C. Tian and X. Hou, *Joule.* **4**, 2646-2660 (2020).<https://doi.org/10.1016/j.joule.2020.09.010>

38. V. Thompson, Toshiba claims 16.6% efficiency for polymer film-based large-area perovskite solar module, 2023, <https://www.pv-magazine.com/2023/08/25/toshiba-claims-16-6-efficiency-for-polymer-film-based-large-area-perovskite-solar-module/>.

39. Toshiba, Toshiba’s Polymer Film-Based Perovskite Large-Area Photovoltaic Module Reaches Record Power Conversion Efficiency of 15.1%, 2021, <https://news.toshiba.com/press-releases/press-release-details/2021/Toshibas-Polymer-Film-Based-Perovskite-Large-Area-Photovoltaic-Module-Reaches-Record-Power-Conversion-Efficiency-of-15.1/default.aspx>.

40. Toshiba, Toshiba’s Perovskite Photovoltaic Modules have the World’s Highest Solar Cell Power Conversion Efficiency, 2018, <https://www.global.toshiba/ww/news/corporate/2018/08/tp0901.html>.

41. E. Bellini, Panasonic testing BIPV glass based on perovskites, 2023, <https://www.pv-magazine.com/2023/09/05/panasonic-testing-bipv-glass-based-on-perovskites/>.

42. A. Extance, *Nature.* **570**, 429-432 (2019).<https://doi.org/10.1038/d41586-019-01985-y>

43. G. Overton, Saule prints flexible perovskite solar modules with consistent 10% efficiency, 2019, <https://www.laserfocusworld.com/detectors-imaging/article/14035450/saule-prints-flexible-perovskite-solar-modules-with-consistent-10-efficiency>.

44. SOLARONIX, Solaronix Achieves Major Breakthrough Toward Perovskite Solar Cell Industrialization, 2016, <https://www.solaronix.com/news/solaronix-achieves-major-breakthrough-toward-perovskite-solar-cell-industrialization/>.

45. J. Zhao, CHINA ADVANCES TO GW-SCALE MASS PRODUCTION OF PEROVSKITE SOLAR CELLS, 2024, <https://www.mitsui.com/mgssi/en/report/detail/__icsFiles/afieldfile/2024/08/13/2407_t_zhao_e.pdf>.

46. R. Peleg, SolaEon claims 21.95% efficiency of monolithic perovskite tandem solar cells, 2024, <https://www.perovskite-info.com/solaeon-claims-2195-efficiency-monolithic-perovskite-tandem-solar-cells>.

47. PVTIME, 21.5%! Mellow Energy Sets Efficiency Record for Flexible Perovskite Module, 2023, <https://www.pvtime.org/21-5-mellow-energy-sets-efficiency-record-for-flexible-perovskite-module/>.

48. E. Bellini, Singaporean startup achieves 22.6% efficiency for perovskite solar module, 2024, <https://www.pv-magazine.com/2024/07/17/singaporean-startup-achieves-22-6-efficiency-for-perovskite-solar-module/>.

49. E. Bellini, Italian startup develops 20.7%-efficient mini perovskite solar modules with 99.6% geometrical fill factor, 2024, <https://www.pv-magazine.com/2024/06/03/italian-startup-develops-20-7-efficient-mini-perovskite-solar-modules-with-99-6-geometrical-fill-factor/>.

50. SunFLex, 17.4%SunFLex´s large-area flexible perovskite efficiency breaks the record again!, 2024, <https://www.sflex.cn/newsinfo/7294656.html>.

51. Rollshine, Efficiency breakthrough丨Rollshine's flexible perovskite module obtains SCM authoritative certification, 2024, <https://mp.weixin.qq.com/s/d6cQFncy4UysShLbR7cWFg>.

52. Solliance, Solliance sets world record for roll-to-roll produced perovskite-based solar cells with a stabilized efficiency of 12,6%, 2024, <https://www.solliance.eu/2017/solliance-sets-world-record-for-roll-to-roll-produced-perovskite-based-solar-cells-with-a-stabilized-efficiency-of-126/>.

53. NREL, Best Research-Cell Efficiency Chart, <https://www.nrel.gov/pv/interactive-cell-efficiency.html>.

54. LONGi, LONGi announces new conversion efficiency of 33.5% for its silicon-perovskite tandem solar cells at Intersolar Europe 2023, 2023, <https://www.longi.com/en/news/new-conversion-efficiency/>.

55. LONGi, LONGi announces the new efficiency of 31.8% for perovskite/crystalline silicon tandem solar cells based on commercial CZ silicon wafers, 2023, <https://www.longi.com/en/news/new-efficiency-of-solar-cells/>.

56. R. Peleg, LONGi announces new world record efficiency of 30.1% for commercial M6 size wafer-level silicon-perovskite tandem solar cells, 2024, <https://www.perovskite-info.com/longi-announces-new-world-record-efficiency-301-commercial-m6-size-wafer-level>.

57. V. Thompson, Oxford PV sets 28.6% efficiency record for full-size tandem cell, 2023, <https://www.pv-magazine.com/2023/05/24/oxford-pv-sets-28-6-efficiency-record-for-full-size-tandem-cell>.

58. V. Thompson, Fraunhofer ISE, Oxford PV produce 25%-efficient perovskite-silicon tandem PV module, 2024, <https://www.pv-magazine.com/2024/01/31/fraunhofer-ise-announces-25-efficient-perovskite-silicon-tandem-photovoltaic-module/>.

59. J. Gifford, Oxford PV unveils 26.9% perovskite tandem module efficiency record, 2024, <https://www.pv-magazine.com/2024/06/19/oxford-pv-presents-26-9-cell-efficiency-record-at-intersolar-europe-2024/>.

60. PVTIME, Trina Solar Cancels Fundraising for 20GW Solar Cell and 10GW Module Projects, 2024, <https://www.pvtime.org/trina-solar-cancels-fundraising-for-20gw-solar-cell-and-10gw-module-projects/>.

61. V. Thompson, US startup developing perovskite-silicon tandem panels with 26% efficiency, 2024, <https://www.pv-magazine.com/2024/01/19/tandem-pv-raises-6-million/>.

62. V. Thompson, U.S. startup develops 28%-efficient perovskite-silicon tandem solar module, 2024, <https://www.pv-magazine.com/2024/08/05/u-s-startup-develops-28-efficient-perovskite-silicon-tandem-solar-module/>.

63. S. Global, Microquanta claims record 26.63% efficiency on 4-terminal perovskite-Si tandem module, 2024, <https://www.solarbeglobal.com/Microquanta-claims-record-26-63-efficiency-on-4-terminal-perovskite-Si-tandem-module/>.

64. The 4th tandemPV Workshop will be held from June 26-28, 2024, in Amsterdam, the Netherlands., <https://tandempv.conexio-pse.de/>.

65. 3Sun, Tandem 3SUN technology, a new efficiency record at 28.7%, 2024, <https://www.3sun.com/en/search-news/news/2024/06/Tandem-3SUN-technology-a-new-efficiency-record>.

66. R. Peleg, RenShine Solar announces 24.5% efficiency for all-perovskite tandem cell module, 2022, <https://www.perovskite-info.com/renshine-solar-announces-245-efficiency-all-perovskite-tandem-cell-module>.
